# Role of Theophylline in Management of Bradycardia Secondary to High Cervical Spinal Cord Injury in a Seven-Year-Old Child: Case Report and a Review of Literature

**DOI:** 10.7759/cureus.10941

**Published:** 2020-10-14

**Authors:** Farida Karim, Philip Chang, Carrie Garrison, Matthew Steiner

**Affiliations:** 1 Pediatrics, University of Florida, Studer Family Children’s Hospital at Sacred Heart, Pensacola, USA; 2 Cardiology, University of Florida Health Shands Children’s Hospital, Congenital Heart Center, Gainesville, USA; 3 Pediatric Critical Care, University of Florida, Studer Family Children’s Hospital at Sacred Heart, Pensacola, USA; 4 Pediatric Cardiology, University of Florida, Studer Family Children’s Hospital at Sacred Heart, Pensacola, USA

**Keywords:** cervical spinal injury, bradycardia, asystole, theophylline, pacemaker

## Abstract

High-level cervical spinal cord injury (SCI) frequently leads to the development of severe sinus bradycardia and asystole. Conventionally, owing to their chronotropic effects, medical management has largely relied on the use of atropine and/or infusion of pressors such as epinephrine or dopamine as the first-line treatment. However, for severe symptomatic events refractory to medical therapy, cardiac pacemaker implantation may be required. In light of the limited data, found in the adult literature, use of methylxanthines such as theophylline has been suggested for the treatment of bradycardia or asystole in the setting of cervical SCI, but to our knowledge, this treatment approach has not been reported in very young children. We present a case of medical management of bradycardia-asystole episodes in a seven-year-old child who sustained cervical SCI after a motor vehicle accident (MVA). His clinical course was complicated by frequent episodes of symptomatic sinus bradycardia progressing to asystole. Episodes were responsive to atropine, but his events were recurrent and feared to be life-threatening if unobserved, and so pacemaker implantation was being considered. In the hope of averting the need for pacemaker implantation, he was started on enteral theophylline, with blood level monitoring and had remained in normal sinus rhythm without recurrence of severe bradycardic or asystole events for a latent period of 74 days. Subsequently, however, he underwent pacemaker placement.

## Introduction

Bradycardia and cardiac arrest are common complications in cervical spinal cord injury (SCI). Bradycardia has been reported in almost all of these cases and asystole in 15% [1]. Disruption of supraspinal sympathetic afferent impulses to the heart leaves unopposed parasympathetic stimuli that may produce severe bradycardia [2]. Cardiac complications are, however, not long-lasting and usually resolve after the acute stage of the injury, usually in six to eight weeks [3]. Management of bradycardia in these settings usually involves taking measures to prevent hypoxia, minimizing tracheal stimulation thereby preventing initiation of vagovagal reflexes, using atropine as needed for bradycardic episodes, placing a transcutaneous pacer in the emergency setting followed by placement of a transvenous pacemaker and, in resistant cases, considering permanent pacemaker placement [1]. However, the literature shows cases where successful obviation of the need to pacemaker placement with pharmacotherapy, with methylxanthines such as theophylline and aminophylline, has been achieved. Therefore, insertion of a cardiac pacemaker traditionally is reserved for cases refractory to pharmaceutical therapy [2].

## Case presentation

A seven-year-old male who was an unrestrained passenger in a motor vehicle accident (MVA) was admitted to the ICU. He sustained significant injuries with resultant quadriplegia and neurogenic shock. Emergent open reduction internal fixation of occiput-atlas dislocation and atlanto-axial dislocation with occiput-C1 and C1-2 arthrodesis was performed.

Post-operatively, he had recurrent symptomatic episodes of bradycardia and asystole. These episodes were usually associated with deep chest suctioning and other manipulation that resulted in hypoxemia, bradycardia (40-50 bpm), and occasional ventricular pauses with lack of escape rhythm. Some of these episodes were associated with syncope and respiratory arrest and required administration of positive pressure ventilation by bag-mask for resuscitation. Bradycardia initially responded to IV administration of atropine. Given the persistence of symptomatic bradycardia and asystolic pauses, pacemaker placement was considered. However, given potential SCI-associated bradycardia to resolve within a few weeks beyond the acute phase of injury [4], medical management with theophylline was trialed in hope of avoiding the need for invasive therapy.

He was started on a low dose of theophylline (3.5 mg/kg four times a day). Serum theophylline levels (STL) were followed with a goal of less than 20 mcg/ml to prevent toxic effects. After the initiation of therapy, a reduction in frequency of bradycardia episodes was observed and heart rate (HR) steadily increased up to 100 bpm. Bradycardia associated with suctioning and changes of ventilator tubing still occurred, prompting further dosing increase.

**Figure 1:**
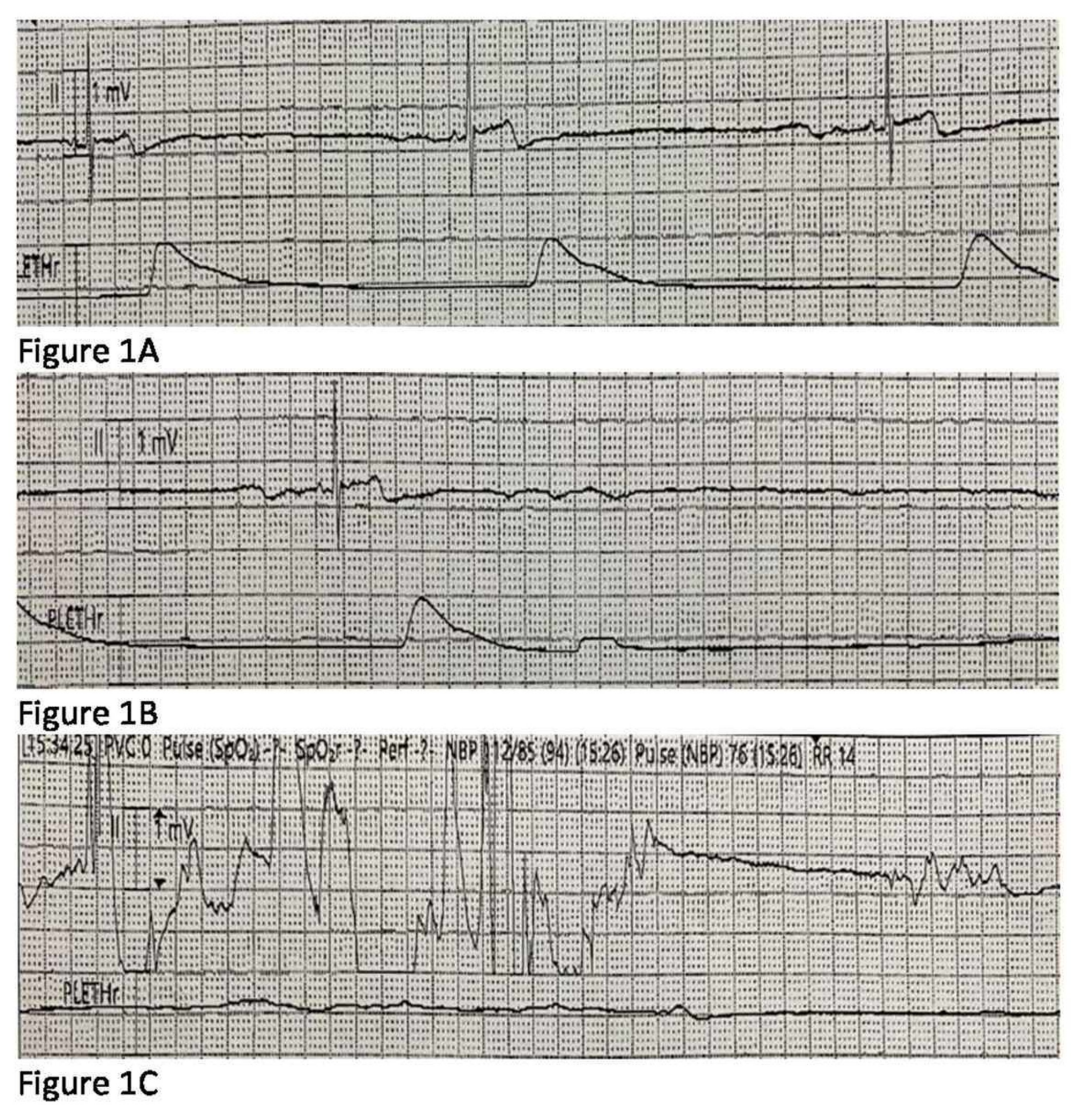
A-C Event recording showed two consecutive 8-second sinus pauses

**Figure 2:**
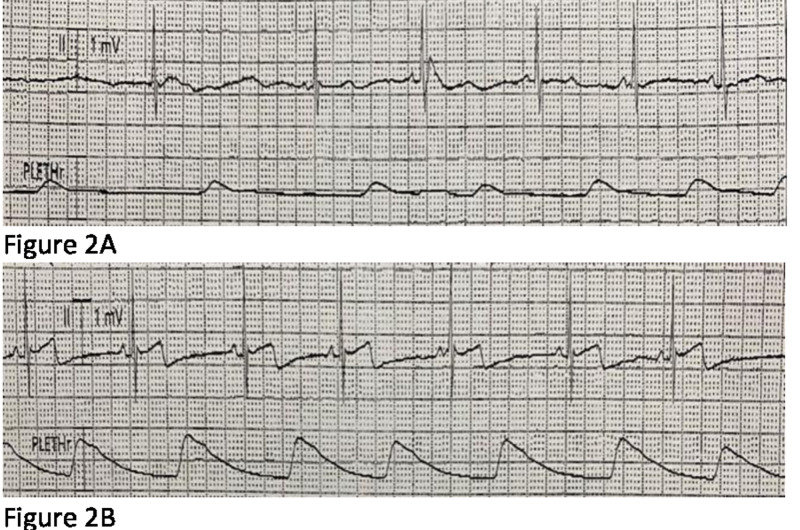
A-B Event recording showed sinus pauses (Figure 1 A-C) followed by very slow sinus bradycardia at 20 bpm before eventual rate normalisation

After four weeks of therapy, his bradycardia events were substantially decreased in frequency and HR ranged between 80-140 bpm. Theophylline dosing was consistent for two weeks (6.3 mg/kg = 150 mg three times a day) with stable STL within the goal range without toxicity. He remained clinically stable for another month but subsequently developed hydrocephalus requiring ventriculoperitoneal shunt placement. Following surgery, his HR began to trend down and 11 days after surgery (and 74 days after initiation of theophylline therapy), he experienced an asystole episode while being moved from his bed to chair for physical therapy. Event recording showed two consecutive 8-second sinus pauses followed by very slow sinus bradycardia at 20 bpm before eventual rate normalization (Figure 1 A-C and Figure 2 A-B).

Given recurrence of profound bradycardia and asystolic pauses despite theophylline therapy, permanent pacemaker implantation was recommended. A Medtronic transvenous dual chamber permanent pacemaker (PPM; Medtronic, Minneapolis, MN, USA) was implanted and theophylline was discontinued. Conventional programming to permit native conduction system functionality and to minimize unnecessary ventricular pacing was implemented along with rate drop response protection. If an absolute ventricular rate drop size of 25 bpm or decline in ventricular rate to 60 bpm were to occur within a 20-second detection window, the device was set to respond with a two-minute intervention of pacing at 100 bpm. Subsequent interrogation has revealed several episodes of rate drop response activation over a four-month follow-up period since device implantation.

## Discussion

This case report describes the management of autonomic mediated bradycardia following high SCI in seven-year-old child using oral theophylline. To our knowledge this is the first case reported for a child younger than 16 years of age who was managed with theophylline as primary therapy to treat symptomatic bradycardia/asystole events after a high SCI as well as the longest latent period (74 days) of clinical effect with this medication for this condition.

Severe bradycardia with pause-dependent ventricular arrhythmias and cardiac arrest is a well-documented complication of acute injury to the cervical spinal cord. This life-threatening bradyarrhythmic state is attributed to an imbalance in the autonomic nervous system (ANS) resulting from dissociation of the parasympathetic from the sympathetic responses during the early stage of spinal shock [1]. The disruption of supraspinal sympathetic pathways, and the resultant parasympathetic dominance, as consequence of cervical SCI is the major cause of cardiovascular instability [5]. Typically, the frequency and severity of bradycardic events are directly related to the severity of the cervical SCI [6]. In addition, the level of the injury is also associated with poor long-term prognosis. Studies suggest that injury above the fifth cervical vertebra (C5) is associated with more cardiovascular complications than other levels of SCI [7].

The goal for providing treatment and interventions for survivors of SCI is directed towards the prevention and acute treatment of bradyarrhythmias [8]. The mechanism responsible for nearly all relevant episodes of severe bradycardia and asystole involves unopposed activity of the parasympathetic ANS, therefore, acute pharmacologic blockade of muscarinic receptors with intravenous atropine is a nearly universally implemented initial treatment as per Advanced Cardiac Life Support guidelines [8].

In the adult literature, numerous case series have been reported that described the use of methylxanthines as preventive therapy for dangerous bradyarrhythmias [5]. Methylxanthines directly antagonize the effects of endogenous adenosine at a variety of receptor subtypes, including the A1A receptor found in the sinoatrial node and AV node [9]. Importantly, methylxanthines also exert beneficial effects on diaphragmatic muscle strength, which is often desirable in patients with high-level SCI [8].

Pharmacologic measures are not always sufficient and pacemaker placement may be necessary. The reported number of SCI patients requiring a pacemaker varies from nine to 17% [10,11].

In the absence of clear-cut guidelines for the management of bradyarrhythmias associated with high SCI, it is reasonable to use atropine and intravenous chronotropic agents as acute, first-line strategies, but their failure to prevent severe recurrent bradycardia has been well documented. For recurrent episodes, preventive pharmacologic therapy with methylxanthines appears to be safe and should be considered as a potentially effective non-invasive therapy during the acute phase of recovery from SCI and prior to consideration of permanent pacemaker implantation [8].

## Conclusions

Theophylline appeared to be beneficial for decreasing severe recurrent bradycardia and asystole events in this seven-year-old patient suffering an acute high cervical SCI.
